# New phthalimide analog ameliorates CCl_4_ induced hepatic injury in mice via reducing ROS formation, inflammation, and apoptosis

**DOI:** 10.1016/j.sjbs.2021.07.014

**Published:** 2021-07-14

**Authors:** Bishoy El-Aarag, Alshaimaa Attia, Magdy Zahran, Ali Younes, Ehab Tousson

**Affiliations:** a Biochemistry Division, Chemistry Department, Faculty of Science, Menoufia University, Shebin El-Koom 32512, Egypt; b Department of Chemistry, Faculty of Science, Menoufia University, Shebin El-Koom 32512, Egypt; c Zoology Department, Faculty of Science, Tanta University, Tanta, Gharbia, Egypt

**Keywords:** Thalidomide, Liver injury, Phthalimide analog, Oxidative stress, Apoptosis, Inflammation

## Abstract

The present study aimed, for the first time, to examine the biochemical effects of new phthalimide analog, 2-[2-(2-Bromo-1-ethyl-1H-indol-3-yl) ethyl]-1H-isoindole-1,3(2H)-dione, compared to thalidomide drug against liver injury induced in mice. Carbon tetrachloride was intraperitoneal injected in mice for 6 consecutive weeks at a dose of 0.4 mL/kg twice a week for liver injury induction. Histopathological examination, levels of malondialdehyde, nitric oxide, and antioxidant enzymes were determined. Additionally, the protein levels of vascular endothelial growth factor, proliferating cell nuclear protein, tumor necrosis factor-alfa, nuclear factor kappa B-p65, B-cell lymphoma-2, and cysteine-aspartic acid protease-3 were determined. Results revealed that the treatment with phthalimide analog improved the detected liver damage and presented an obvious antioxidant activity through decreasing malondialdehyde and nitric oxide levels accompanied by increasing the levels of the antioxidant enzymes. Furthermore, the analog exhibited an effective inhibitory activity towards the studied protein expressions in liver tissues. Moreover, the B-cell lymphoma-2 protein level was increased while the cysteine-aspartic acid protease-3 level was suppressed after the treatment with phthalimide analog. Together, these results propose that phthalimide analog can ameliorate carbon tetrachloride-induced liver injury in mice through its potent inhibition mediating effect in oxidative stress, inflammation, and apoptosis mechanisms.

## Graphical abstract

A proposed mechanism of action for phthalimide analog ameliorating CCl_4_–induced liver injury in mice.

**Figure:**
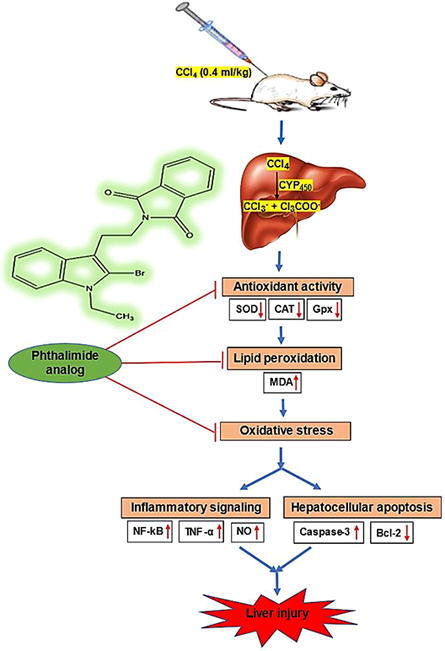

## 1 Introduction

The alteration in cellular oxidation–reduction balance and the high level of reactive oxygen species (ROS) production initiates oxidative stress. It is believed to be a crucial risk factor in the development of liver disease (Uchida et al., 2020). A common experimental liver injury model was established via intoxication with carbon tetrachloride (CCl_4_). Cytochrome P450 catalyzed the conversion of CCl_4_ to a trichloromethyl radical (∙CCl_3_). In the presence of molecular oxygen, ∙CCl_3_ transformed to trichloromethyl peroxy free radicals (CCl_3_OO∙) which interferes with microsomal membranes leading to lipid peroxidation, membrane impairment and consequently hepatocellular injury (Jeong et al., 2020). Antioxidative enzymes are scavengers to ∙CCl_3_ and lipid peroxy radicals in CCl_4_-induced oxidative stress in hepatocytes (Li et al., 2015). Superoxide dismutase (SOD), catalase, and glutathione peroxidase (GPx) are complicated in the protection towards ROS (He et al., 2017).

Nuclear factor kappa B (NF-kB) is a heterodimer protein complex consisted of many subunits including P65. It is associated with inhibitory protein kappa Bα (IkBα) in the cytosol. CCl_4_-induced oxidative stress could stimulate the phosphorylation then degradation of IkBα causing the phosphorylation of NF-kB-p65 and translocation to the nucleus (Lingappan, 2018). In the nucleus, NF-kB-p65 regulates the expression of inducible inflammatory mediators including tumor necrosis factor-alpha (TNF-α) and nitric oxide (NO) (Liu et al., 2017) through its binding to specific DNA sequences (Sun et al., 2015).

Apoptosis, programmed cell death, plays a physiological vital role in embryogenesis and tissue remodeling through removal of unwarranted cells. In spite of the etiology, liver injury is characterized by augmented hepatocyte apoptosis. Apoptosis is involved in an extensive variety of acute and chronic diseases such as liver diseases (Shojaie et al., 2020). Thus, the discovery of new potent agents that complicated in the apoptosis process may offer new opportunities for the treatment of liver diseases.

Heterocyclic phthalimide (isoindoline-1,3-dione) derivatives are biologically active pharmacophores and exhibited an important role in drug discovery (Wang et al., 2013, Si et al., 2016). It possesses numerous biological properties such as anti-cancer (Kamal et al., 2013), antioxidant (Rajasekaran et al., 2011) and anti-inflammatory (Pophale and Deodhar, 2010). Thalidomide was synthesized in 1950s with defined chemical structure that consists of phthalimide and glutarimide moieties. Nevertheless, its reported teratogenicity it was confirmed to possess anti-angiogenic and anti-cancer activities (Zhang and Luo, 2018). Thalidomide also was used in the treatment of multiple myeloma and malignant B cell lymphoma (Sinit et al., 2019). Numerous N-substituted phthalimide-based drug analogs with potent anticancer therapeutic activities were stated (Guirgis et al., 2010, El-Aarag et al., 2014, El-Aarag et al., 2017). Also, recent phthalimide-indole derivatives with several biodiversity were reported (Zahran et al., 2018).

The current study was planned to assess the *in vivo* mechanism of action of new phthalimide derivative, in comparison with thalidomide drug, in mice with liver injury induced by CCl_4_. With the aim of determine the possible therapeutic mechanisms and providing new treatment agents for CCl_4_ induced liver damage.

## 2 Materials and methods

### 2.1 Chemicals

Thalidomide was used as a phthalimide-based reference drug. Dimethyl sulfoxide (DMSO; 99.9%; Sigma, USA) was used as a vehicle to dissolve phthalimide analog and thalidomide.

### 2.2 Preparation method of phthalimide analog

**Scheme 1:**
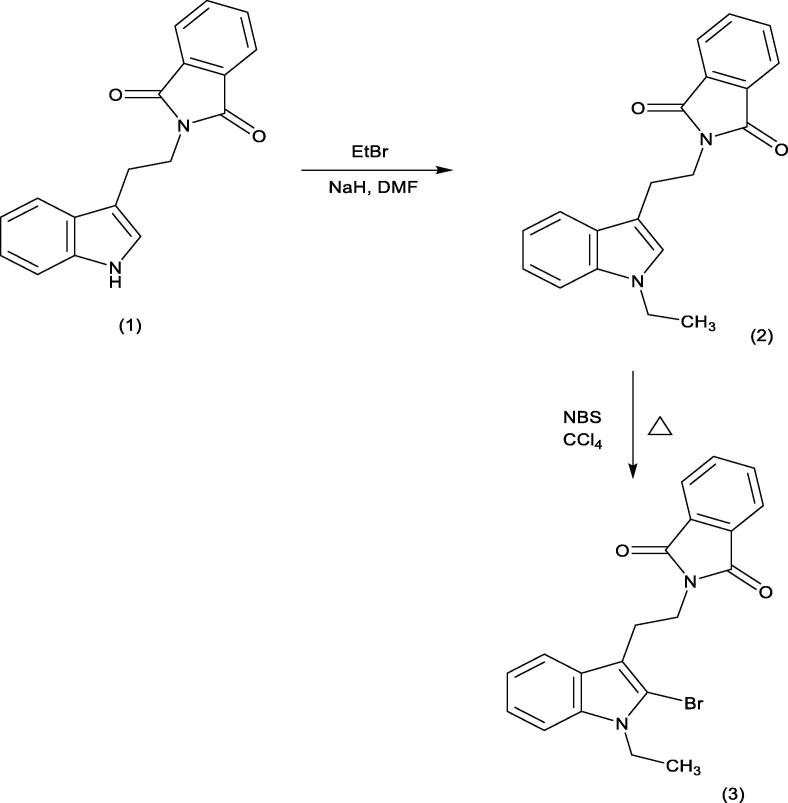
Preparation method of phthalimide analog.

Phthalimide analog was prepared according to Zahran et al., 2018. In brief, a solution of isoindole (1) in dry dimethylformamide (DMF), sodium hydride (NaH) was added and kept stirring for 30 min, then ethyl bromide was added. The reaction mixture was heated under reflux for 1 h. The product was extracted and the organic layer was dried, filtered off, and purified to give the derivative (2). N-Bromosuccinimide (NBS) was added and the reaction mixture was reflux for 1 h. Then, the formed precipitate was filtered off, the solvent was evaporated, and the residue was recrystallized to give phthalimide analog (3) as shown in Scheme 1.

### 2.3 Animals

The current study included 40 adult male Swiss albino mice with eight weeks old and average 25 g body weight. Mice were administrated food and water *ad libitum*. Sterilized cages were used to keep mice using 12/12 h light/dark round in addition to controlled temperature. Laboratory animals care and use were applied depending on the guidelines of the Institutional Animal Care Committee (Reg. No. SFMU-30918) which compatible with the national institute of health.

### 2.4 Induction of liver injury

Liver injury was established in mice mediated by the action of CCl_4_. CCl_4_ (99.9%) was obtained from Sigma Aldrich (St Louis, Missouri, USA) and intraperitoneal (IP) injected in mice with a dose of 0.4 mL/kg body weight diluted in olive oil twice a week for 6 consecutive weeks (El-Aarag et al., 2019, El-Aarag et al., 2019a).

### 2.5 Mice groups

The current study was included four groups (ten mice/group) and classified as the following:

**Group 1**: (Normal control group) mice administrated 100 µl of olive oil twice a week for 6 consecutive weeks.
**Group 2**: (LI group) mice injected with 100 µl CCl_4_/olive oil twice a week for 6 consecutive weeks followed by IP treated with 100 µl DMSO/H_2_O by ratio (0.7:0.3) for two successive weeks (5 times/week).
**Group 3**: (LI + phthalimide analog) mice injected with 100 µl CCl_4_/olive oil twice a week for 6 consecutive weeks followed by IP treated with 100 µl phthalimide derivative (30 mg/kg body weight) for two successive weeks (5 times/week).
**Group 4**: (LI + Thalidomide) mice injected with 100 µl CCl_4_/olive oil twice a week for 6 consecutive weeks followed by IP treated with 100 µl thalidomide (30 mg/kg body weight) for two successive weeks (5 times/week).

The treated dose of phthalimide analog and thalidomide was selected according to the maximum tolerated dose as previously reported (El-Aarag et al., 2017).

### 2.6 Liver tissues processing

Mice were anaesthetized and sacrificed post the finishing of the treatment period afterward liver tissues were detached and cleaned with saline solution. The lobes of liver tissues were then separated and the left lobes were used in histological and immunohistochemical assays after fixation in neutral buffered formalin (10%).

### 2.7 Liver homogenate preparation

Liver homogenates were prepared from the right lobes of liver tissues. Cold potassium phosphate buffer (50 mM, pH 7.4) was used to prepare 10% liver homogenates then the obtained suspensions were centrifuged for 10 min at 2000 rpm at 4 °C. The supernatants were frozen at −80 °C for the detection of the activity of antioxidant enzymes (SOD, CAT, and Gpx) as well as the apoptosis-related proteins including B-cell lymphoma-2 (Bcl-2) and cysteine-aspartic acid protease-3 (caspase-3).

### 2.8 Measurment of malondialdehyde (MDA) level

Lipid peroxidation was indexed and detected in the form of MDA; therefore, its level in the liver tissue homogenate was spectrophotometrically determined as reported method (Ohkawa et al., 1979) through an MDA assay kit (Bio-diagnostic, Giza, Egypt) as stated by the manufacturer’s instructions.

### 2.9 Measurment of anti-oxidant enzymes activites

The effects of phthalimide derivative on SOD, CAT, and GPx activities in liver tissue homogenate were determined through commercial kits (Bio-diagnostic, Giza, Egypt) according to the manufacturer’s instructions (El-Aarag et al., 2019a).

### 2.10 Measurment of NO level

The level of NO in liver tissue homogenate was measured depending on nitrite concentration and according to Griess method via using a commercial NO assay kit (Bio-diagnostic, Giza, Egypt). Nitric oxide is expressed in the sample as μmol/l (El-Aarag et al., 2014).

### 2.11 Hepatic histopathological assessment

Liver tissues were fixed by 10% formaldehyde followed by embedding in paraffin wax. Five μm thickness sections were allowed to float in water bath and then caught with glass slides. The slides were dried and stained with H&E after that light microscope examination (Bancroft and Stevens, 1996).

### 2.12 Immunohistochemical detection of vascular endothelial growth factor (VEGF), proliferating cell nuclear protein (PCNA), tumor necrosis factor-alfa (TNF-α), and nuclear factor kappa B-p65 (NFkBp65)

The protein expressions of VEGF, PCNA, TNF-α, and NFkB-p65 in liver tissues of normal mice and in that treated with phthalimide analog and thalidomide were detected. Polyclonal rabbit anti-VEGF antibody at 1:100 dilution (Thermo Fisher Scientific, Waltham, MA, USA), poly clonal antibody of TNF-α (Invitrogen, USA), NF-κB-p65 at dilution 1:100 (Rel A, ab-1 rabbit polyclonal, Thermo Fisher Scientific, Waltham, MA, USA) and anti-PCNA (clone PC10; 1:200 Dako, Glostrup, Denmark) were involved in the assay. The percentage of positive cells per total 1000 counted cells in eight high power fields was used to represent the labeling index as previously reported (El-Aarag et al., 2021, El-Saied et al., 2019).

### 2.13 Determination of caspase-3 and Bcl-2 levels

The effect of phthalimide derivative on the levels of caspase-3 and Bcl-2 in liver tissue homogenate of normal and different treated mice groups was quantified using caspase-3 and Bcl-2 ELISA assay kits (Cusabio, Wuhan, China) consistent with the manufacturer's instructions.

### 2.14 Statistical analysis

The results were expressed as mean ± SD. One-way analysis of variance (ANOVA) followed by Tukey’s post-hoc test was used to assess the statistical significance of multiple group comparisons, using GraphPad Prism 6. *p* value low 0.05 considered statistically significant.

## 3 Results

### 3.1 Chemical elucidation of phthalimide analog

**Fig. 1:**
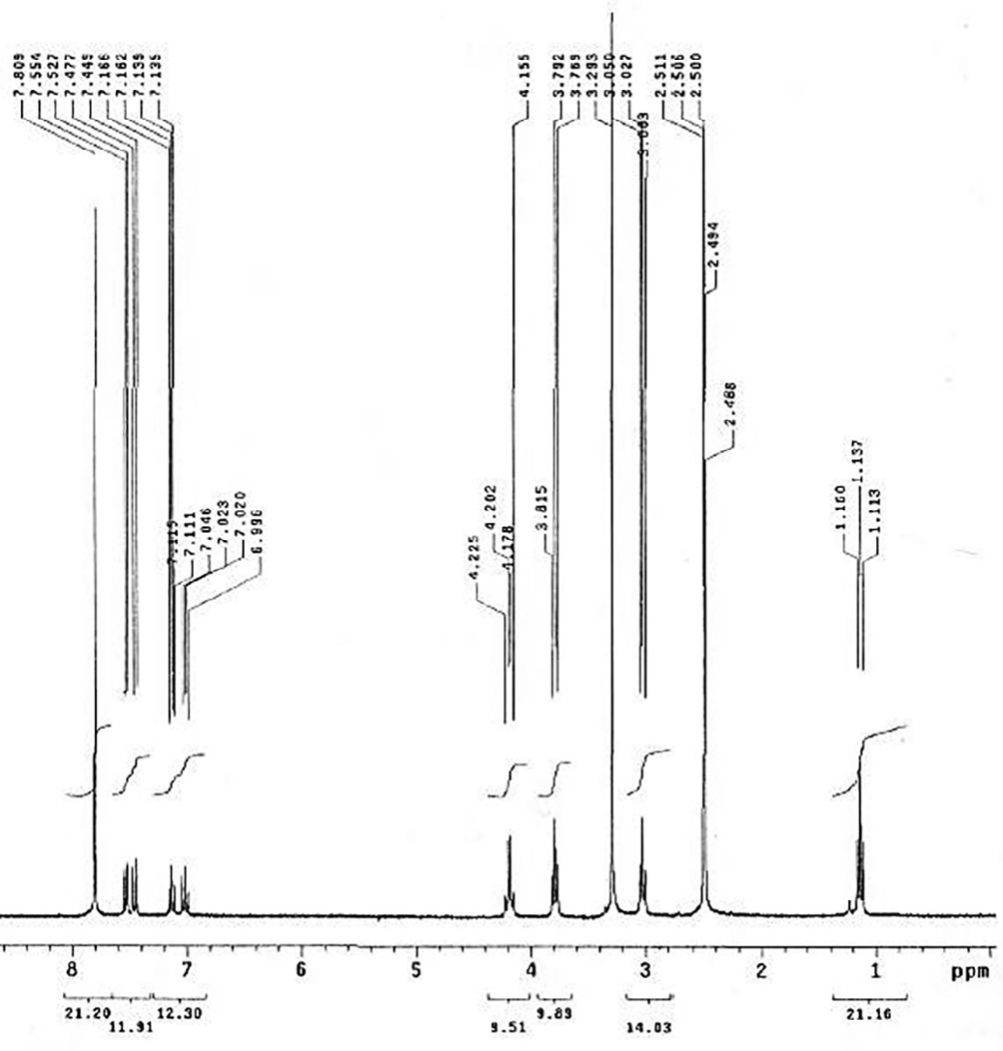
^1^H NMR spectrum of phthalimide analog.

The chemical structure of phthalimide analog was confirmed as previously reported by Zahran et al., 2018. In brief, the ^1^H NMR spectrum of phthalimide analog, 2-[2-(2-Bromo-1-ethyl-1H-indol-3-yl) ethyl]-1H-isoindole-1,3(2H)-dione revealed the following data: ^1^H NMR (DMSO‑*d_6_*, 300 MHz), *δ* ppm: 1.13 (t, *J* = 7.2 Hz, 3H), 3.02 (t, *J* = 7.2 Hz, 2H), 3.79 (t, *J* = 6.9 Hz, 2H), 4.19 (q, *J* = 6.9 Hz, 2H), 7.02 (t, *J* = 7.2 Hz, 1H), 7.13 (t, *J* = 7.2 Hz, 1H), 7.46 (d, *J* = 8.4 Hz, 1H), 7.54 (d, *J* = 8.1 Hz, 1H), 7.81 (s, 4H) as showed in Fig. 1.

**Fig. 2:**
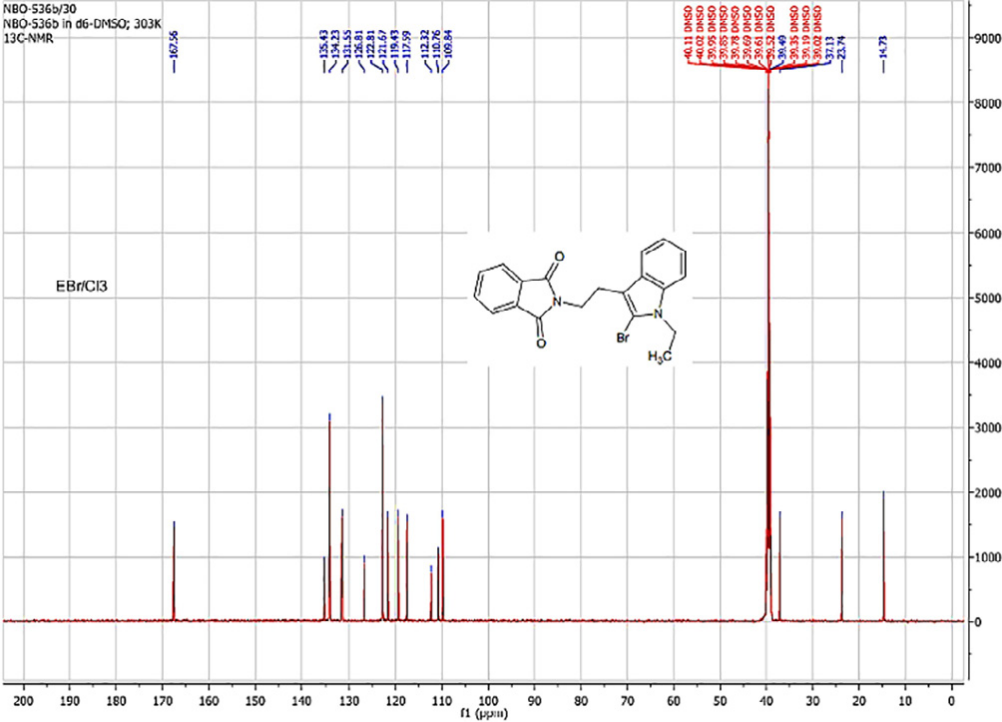
^13^C NMR spectrum of phthalimide analog.

As showed in Fig. 2, the ^13^C NMR spectrum of phthalimide analog gave the following data: ^13^C NMR (DMSO‑*d_6_*, 101 MHz, ppm): *δ* 14.73, 23.74, 37.13, 39.49, 109.84, 110.76, 112.32, 117.59, 119.43, 121.67, 122.81, 126.81, 131.55, 134.23, 135.43, 167.56.

### 3.2 Histopathological examination of liver tissue

**Fig. 3:**
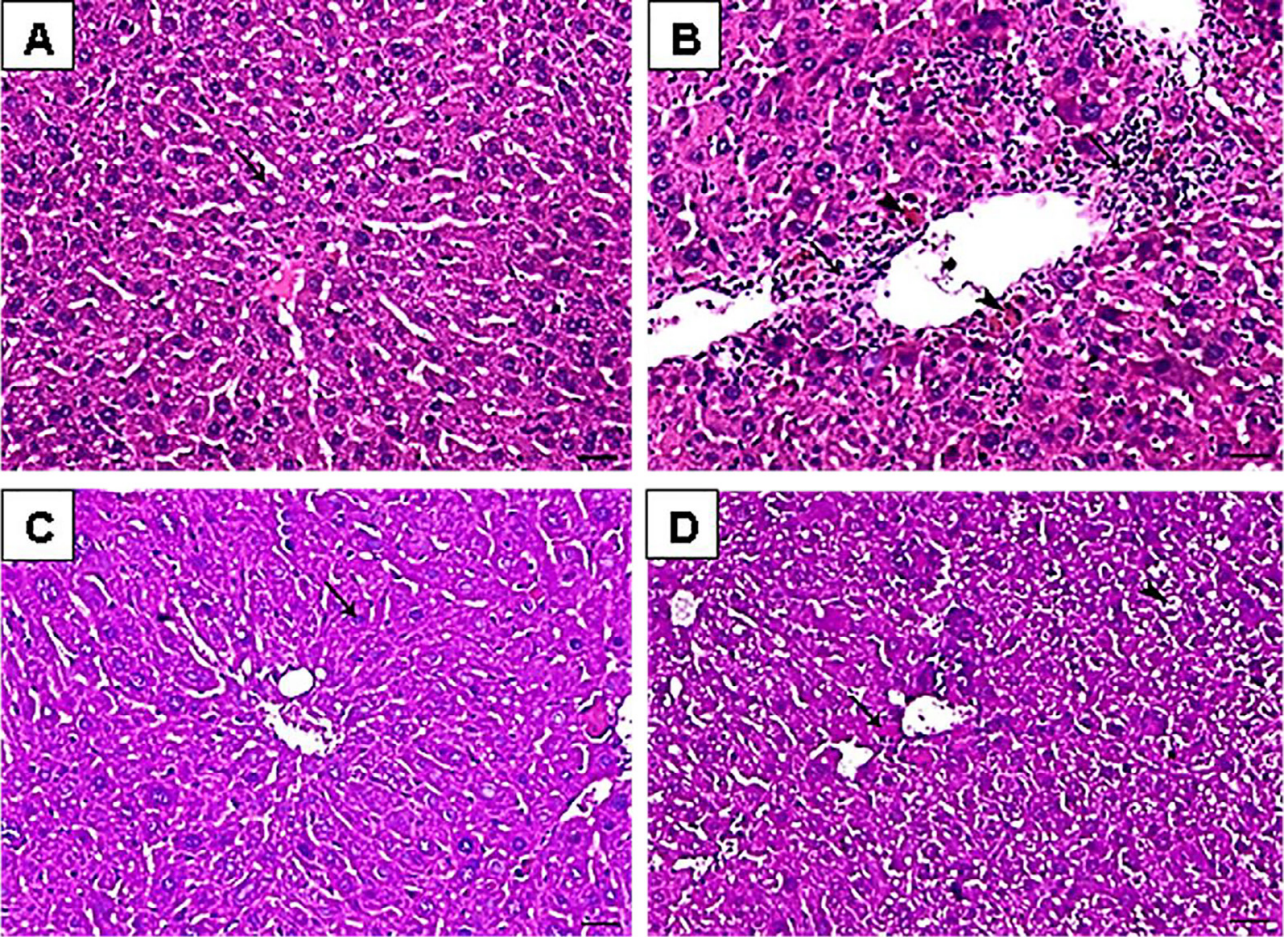
Liver sections from the studied groups stained with Hematoxylin and eosin (H&E). **A:** normal control group. **B:** Liver injury (LI) group. **C:** LI + phthalimide analog group. **D:** LI + thalidomide group. Scale bar = 50 µm.

Histopathological investigation of the liver tissues was revealed in Fig. 3. Normal hepatocytes arranged in cords around the central vein were presented in liver tissues of normal mice (Fig. 3A). Also, there are no detectable congestion, degeneration, necrosis, fibrosis, inflammation, and lesions. Conversely, the liver tissues of LI model group showed multiple histopathological changes demonstrated by centrilobular hepatic necrosis, fibroblastic cells proliferation (arrow), and hemosiderin pigment deposition (arrowhead) as shown in Fig. 3B. Moreover, severe diffuse congestion, severe degeneration, focal necrosis, fibrosis, focal inflammation, and lesions were noticed.

The treatment with phthalimide analog, (30 mg/kg body weight), improved these pathologic changes because there is no detectable fibrosis or necrosis while a mild degree of hepatic degeneration still existing (arrow) (Fig. 3C). In the case of mice treated with thalidomide reference drug, (30 mg/kg body weight), it was found a centrilobular hepatic necrosis (arrow), midzonal, and periportal hepatic vacuolation (arrowhead) as illustrated in Fig. 3D. Also, mild fibrosis and moderate necrosis, congestion, inflammation, and lesion were still existing.

### 3.3 Effect of phthalimide analog on MDA level in liver tissue homogenate

**Fig. 4:**
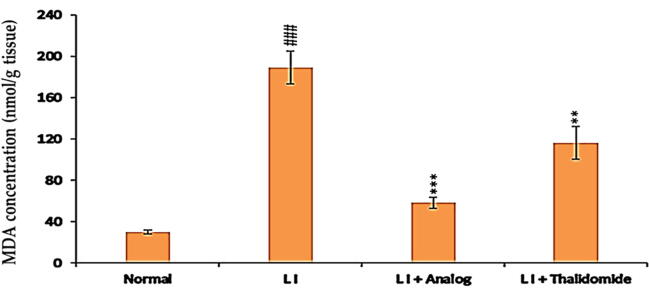
Effect of phthalimide analog and thalidomide on MDA level in liver tissue homogenates. Data are shown in the form of mean ± SD. A ^###^*p* < 0.001 significant different compared to normal group. ** *p* < 0.01 and *** *p* < 0.001 significant different compared to LI model group. MDA: malonaldehyde, LI: liver injury.

MDA is an aldehyde produced during lipid oxidation and usually used as an oxidation marker. Consequently, the anti-oxidation effect of phthalimide analog and thalidomide on MDA levels in liver homogenates was investigated. Results illustrated that MDA level in LI model group was significantly (*p <* 0.001) augmented in relative to that was measured in liver homogenates of normal control mice group. Compared to LI model group, the treated mice with phthalimide analog revealed a significant (*p* < 0.001) decline in MDA level more than that was found in thalidomide group (*p* < 0.01) as presented in Fig. 4.

### 3.4 Effect of phthalimide analog on the antioxidative enzymes’ activities in liver tissue homogenate

SOD, CAT, and GPx exhibited an important protection role against ROS (Kumaravelu et al., 1995). Table 1 showed the effect of phthalimide analog and thalidomide drug on the activities of SOD, CAT, and Gpx enzymes. The activities of antioxidant enzymes in liver tissue homogenate were significantly (*p* < 0.001) diminished in relative to that were found in normal control group. Conversely, compared to LI model group, the treatment with phthalimide analog significantly (*p* < 0.001) increased the reduced SOD, CAT, and Gpx activities more than the effect of thalidomide (Table 1).

**Table 1:** Effect of phthalimide analog on the activities of antioxidant enzymes in liver tissue homogenates.

**Mice Groups**	**SOD** (U/g tissue)	**CAT** (U/g tissue)	**GPx** (U/g tissue)
Normal	37.67 ± 1.53	62 ± 2.03	35.33 ± 2.31
LI	8.33 ± 2.49 ^###^	21.67 ± 2.52 ^###^	13.33 ± 1.27 ^###^
LI + phthalimide analog	34 ± 1.98 ^***^	57.67 ± 2.37 ^***^	26.83 ± 1.04 ^***^
LI + Thalidomide	17 ± 1.78 *	29.33 ± 1.23 *	16.17 ± 0.76 *

Results are shown in the form of mean ± SD. A ^###^*p* < 0.001 significant compared to normal group. * *p* < 0.05, *** *p* < 0.001 significant different compared to LI group. LI: liver injury; SOD: superoxide dismutase; CAT: catalase; Gpx: glutathione peroxidase.

### 3.5 Effect of phthalimide analog on NO level in liver tissue homogenate

**Fig. 5:**
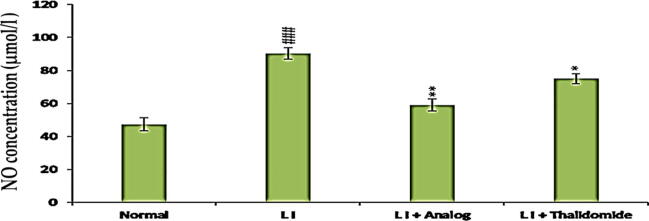
Effect of phthalimide analog on the level of NO level in liver tissue homogenate. Results are shown in the form of mean ± standard deviation. Significantly (^###^*p* < 0.001) compared to normal group. Significantly (* *p* < 0.05, ** *p* < 0.01) compared to LI group**.** LI: liver injury**;** NO: nitric oxide.

NO level in liver tissue homogenates of all studied groups were demonstrated in Fig. 5. A significant (*p* < 0.001) raised level was detected in LI group in comparison to normal control. Alternatively, compared to the LI group, phthalimide analog significantly (*p* < 0.01) diminished the elevated level of NO with effect higher than thalidomide group (*p* < 0.05) as shown in Fig. 5.

### 3.6 Effect of phthalimide analog on VEGF protein level in liver tissues

**Fig. 6:**
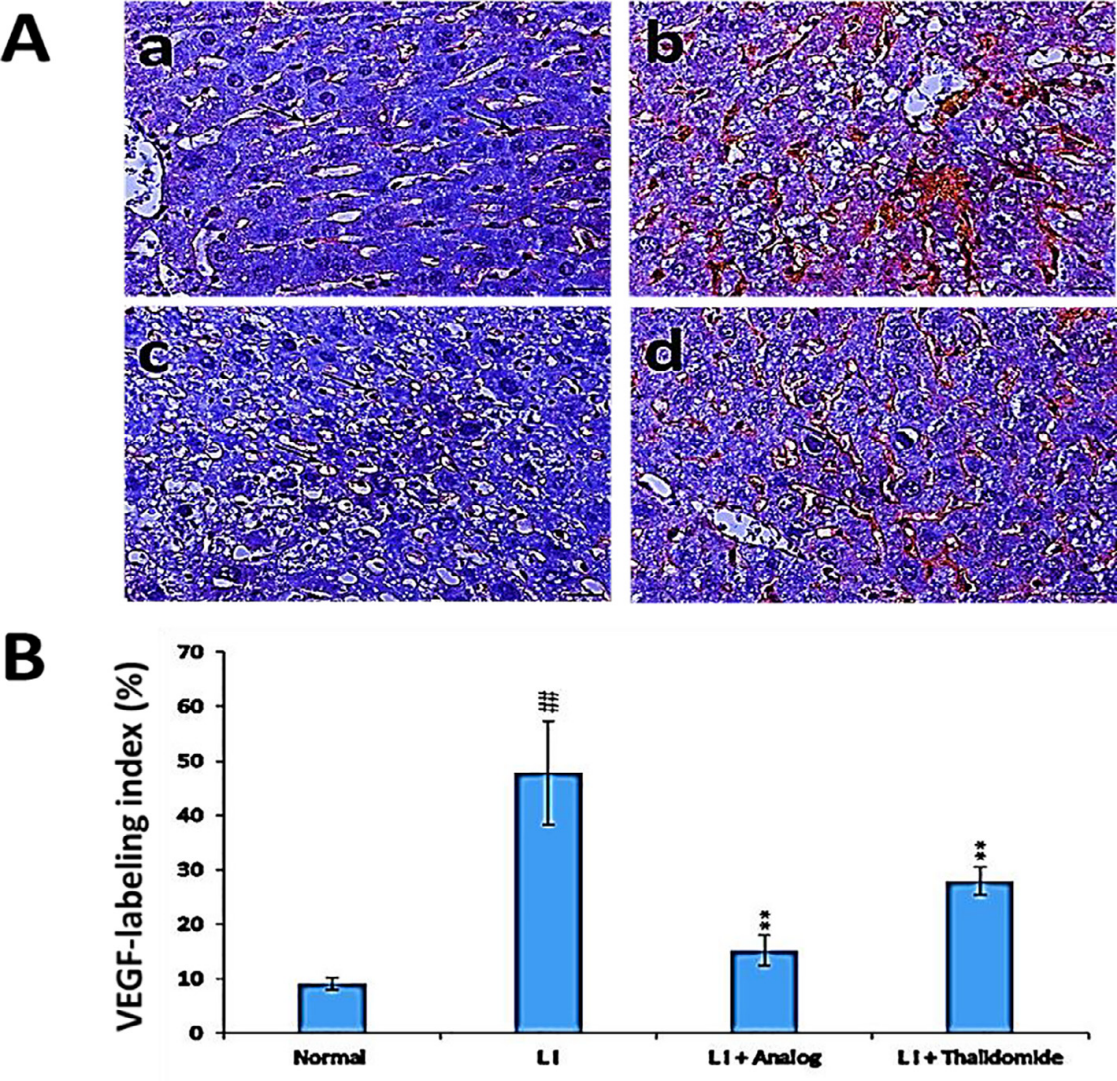
Effect of phthalimide analog on VEGF protein level in hepatic tissues. **A**: Illustrative immunohistochemical photographs where a: normal group, b: liver injury (LI) group, c: LI + phthalimide analog group and d: LI + thalidomide group; scale bar = 50 µm. **B**: Statistical analysis of VEGF labeling index percent. Results are presented as mean ± SD. Significantly (^##^*p* < 0.01) compared to normal group. Significantly (** *p* < 0.01) compared to the LI group. VEGF: vascular endothelial growth factor.

Results revealed a significant (*p* < 0.01) elevation in VEGF level in CCl_4_ induced liver injury group compared to normal control group (Fig. 6). Treatment with phthalimide analog and thalidomide reinstated the protein levels of VEGF compared to LI group. Phthalimide analog showed a significant (*p* < 0.01) inhibition level near to that found in normal control group and more than thalidomide drug.

### 3.7 Effect of phthalimide analog on PCNA protein level in liver tissues

**Fig. 7:**
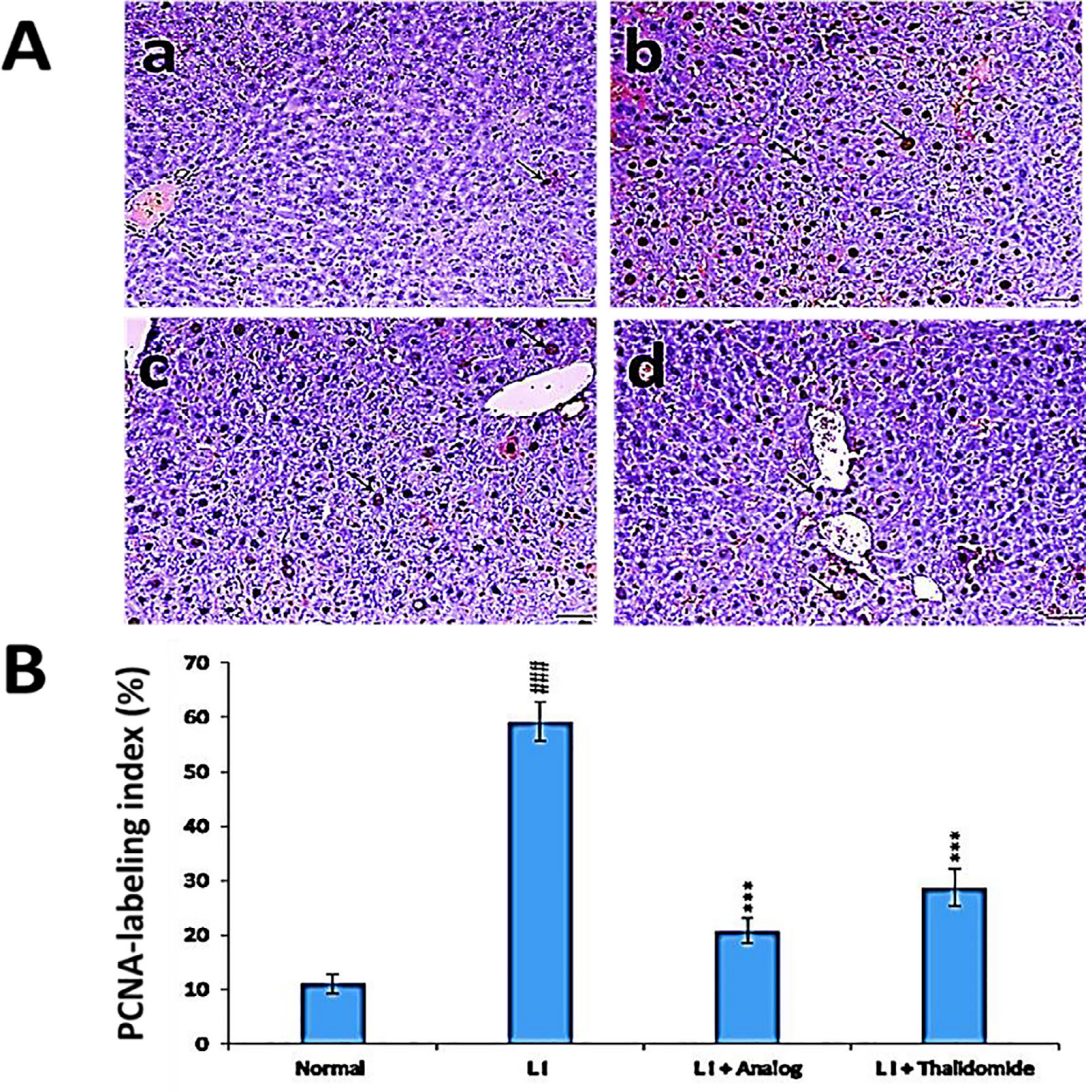
Effect of phthalimide analog on PCNA protein level in hepatic tissues. **A**: Illustrative immunohistochemical photographs where a: normal group, b: liver injury (LI) group, c: LI + phthalimide analog group and d: LI + thalidomide group; scale bar = 50 µm. **B**: Statistical analysis of PCNA labeling index percent. Results are represented as mean ± SD. Significantly (^##^*p* < 0.001) compared to normal group. Significantly (*** *p* < 0.001) compared to the LI group. PCNA: proliferating cell nuclear antigen.

Compared to normal control group, the PCNA level was significantly (*p* < 0.001) elevated in liver tissues after induction of liver injury by CCl_4_ injection. On the other hand, PCNA levels decreased in injured liver tissues of mice treated either with phthalimide analog or thalidomide. Phthalimide analog significantly (*p* < 0.001) reduced PCNA levels, with a potent effect than that detected in thalidomide group (Fig. 7).

### 3.8 Effect of phthalimide analog on TNF-α level in liver tissues

**Fig. 8:**
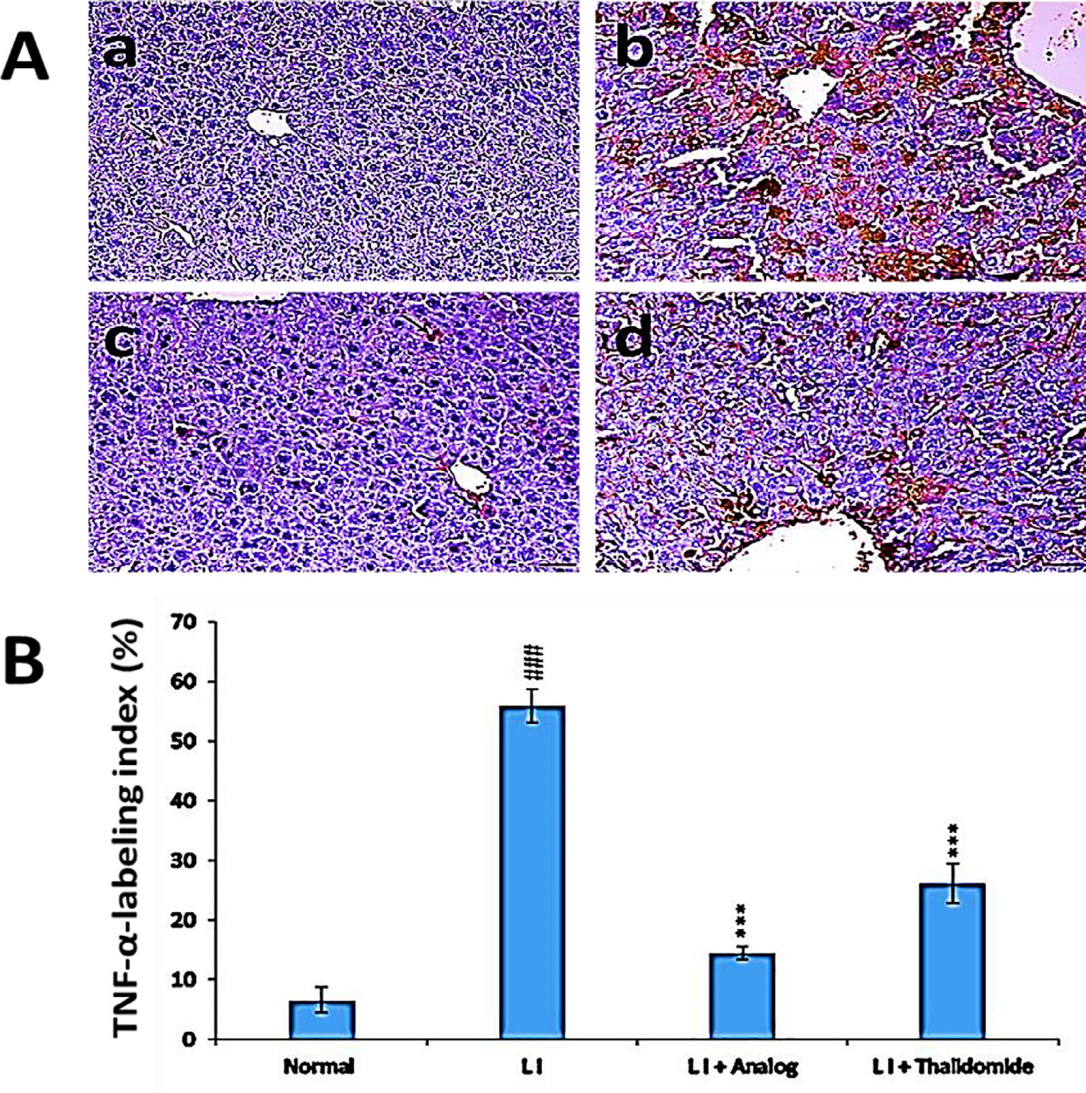
Effect of phthalimide analog on TNF-α protein level in hepatic tissues. **A**: Illustrative immunohistochemical photographs where a: normal group, b: liver injury (LI) group, c: LI + phthalimide analog group and d: LI + thalidomide group; scale bar = 50 µm. **B**: Statistical analysis of TNF-α labeling index percent. Results are represented in the form of mean ± SD. Significantly (^###^*p* < 0.001) compared to normal group. Significantly (*** *p* < 0.001) compared to the LI group. TNF-α: tumor necrosis factor-alfa.

It could be observed from Fig. 8 that there was a significant (*p* < 0.01) decrease in the expression level of TNF-α in liver tissues of LI group in comparison to normal group. The treatment with phthalimide analog or thalidomide can restore the declined TNF-α levels. Phthalimide analog significantly (*p* < 0.001) augmented the declining expression of TNF-α more than thalidomide drug.

### 3.9 Effect of phthalimide analog on NF-ĸB-p65 protein level in liver tissues

**Fig. 9:**
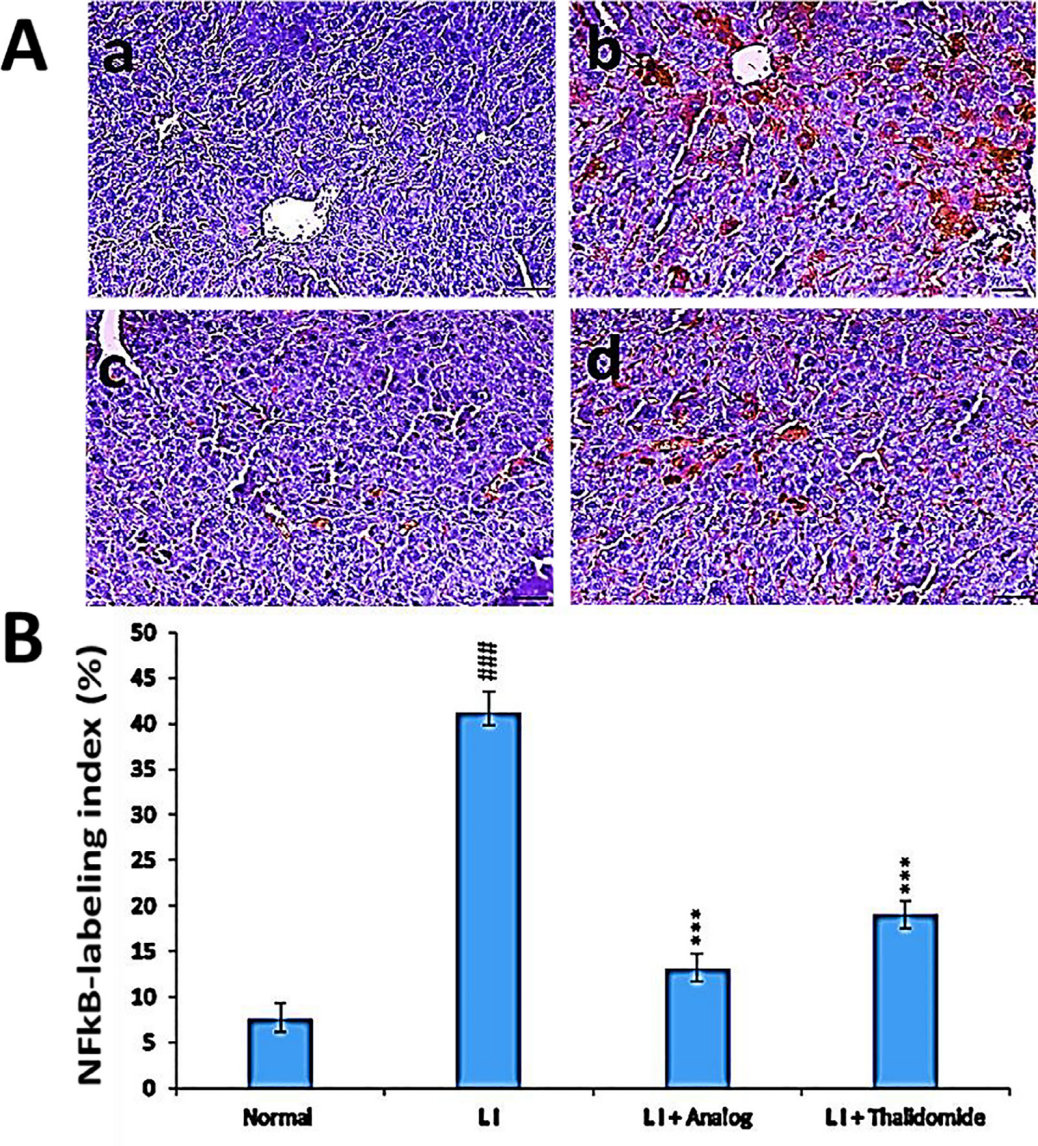
Effect of phthalimide analog on NF-ĸB-p65 protein level in hepatic tissues. **A**: Illustrative immunohistochemical photographs where a: normal group, b: liver injury (LI) group, c: LI + phthalimide analog group and d: LI + thalidomide group; scale bar = 50 µm. **B**: Statistical analysis of NFĸB-p65 labeling index percent. Results are represented as mean ± SD. Significantly (^###^*p* < 0.001) compared to normal group. Significantly (*** *p* < 0.001) compared to the LI group. NF-ĸB-p65: nuclear factor-kappa B-p65.

Results in Fig. 9 presented a significant (*p* < 0.001) elevated level of NF-ĸB-p65 in LI model group due to the action of CCl_4_ compared to normal control group. Treatment with phthalimide analog restored the NF-ĸB-p65 protein level and significantly (*p* < 0.001) decreased the expression level more than that found in thalidomide group in comparison to LI model group.

### 3.10 Effect of phthalimide analog on caspase-3 in hepatic tissues

**Fig. 10:**
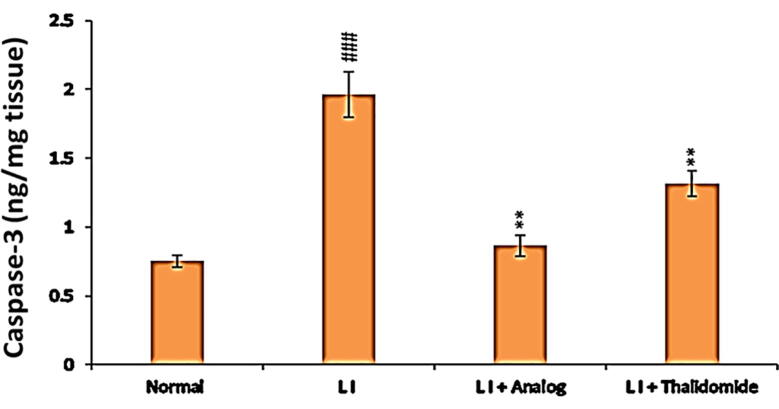
Effect of phthalimide analog on caspase-3 level in hepatic tissues. Data are presented as mean ± SD. Significantly (^###^*p* < 0.001) compared to normal control group. Significantly (** *p* < 0.01) different from LI model group. LI: liver injury; caspase-3: cysteine-aspartic acid protease-3.

Compared to the normal control group, the protein level of caspase-3 was significantly (*p* < 0.001) increased in liver tissues of LI group. On the other hand, in LI + phthalimide analog group the level of caspase-3 was decreased. Phthalimide analog possessed a significant (*p* < 0.001) reduction ability towards pro-apoptotic caspase-3 level more than thalidomide in comparison to the level noticed in the LI group (Fig. 10).

### 3.11 Effect of phthalimide analog on Bcl-2 protein in hepatic tissues

**Fig. 11:**
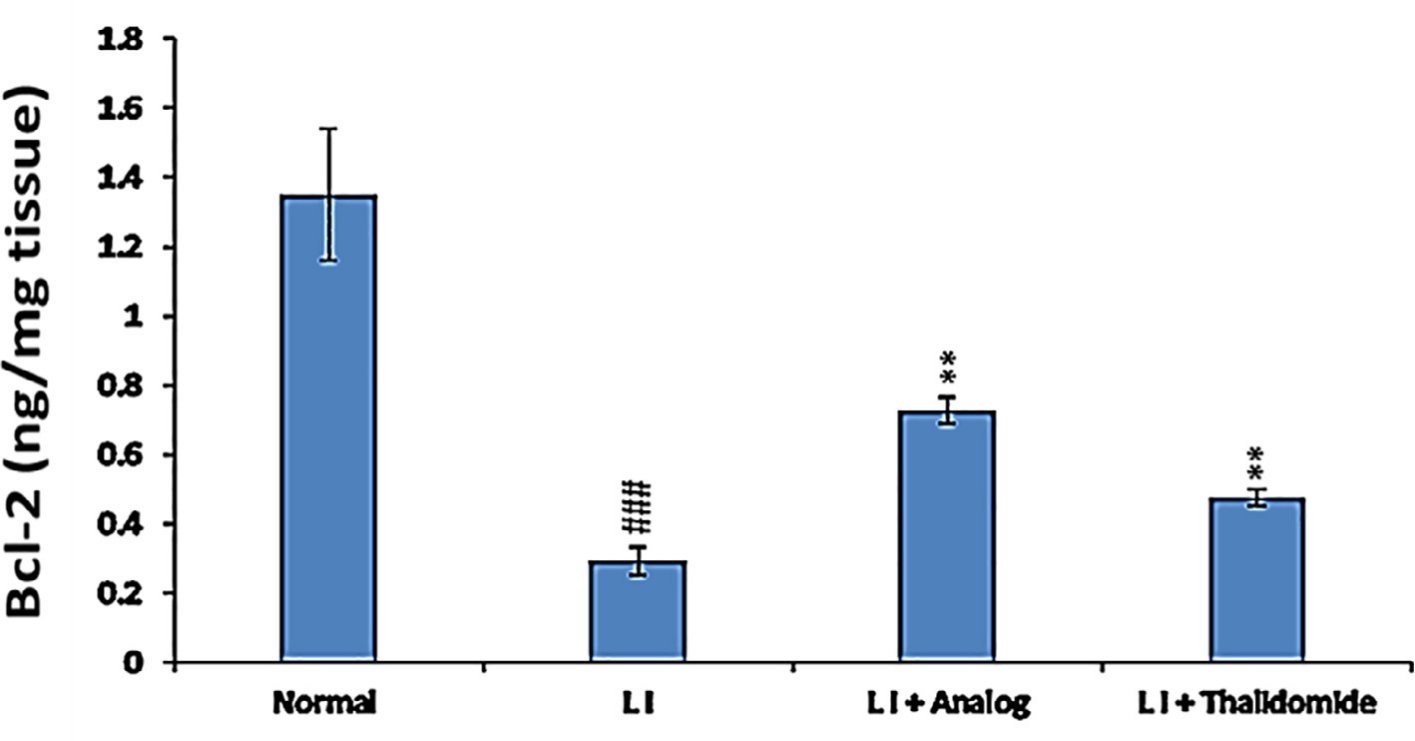
Effect of phthalimide analog on Bcl-2 level in hepatic tissues. Data are presented as mean ± SD. Significantly (^###^*p* < 0.001) compared to normal control group. Significantly (** *p* < 0.01) compared to LI model group. LI: liver injury; Bcl-2: B‐cell lymphoma‐2.

As demonstrated in Fig. 11, the protein level of Bcl-2 was significantly (*p* < 0.01) diminished in liver tissues of LI mice group, compared to the normal control group. Conversely, mice with LI treated with phthalimide analog showed an improvement in the level of anti-apoptotic Bcl-2 as a significant (*p* < 0.001) induction level was detected with pronounced effect more than thalidomide compared to LI group (Fig. 11).

## 4 Discussion

The present study aimed to examine, for the first time, the *in vivo* therapeutic effect and possible mechanisms of new phthalimide analog compared to thalidomide drug towards liver injury model established by CCl_4_ in mice.

The histopathological examination of the stained liver sections from LI group showed centrilobular hepatic necrosis and fibroblastic cells proliferation. Also, severe diffuse congestion, severe degeneration, focal necrosis, fibrosis, focal inflammation and lesions were clearly noticed. These findings are compatible with the previously published studies (Wang et al., 2015, Zhang et al., 2018, Wang et al., 2019). Conversely, compared to LI model group, the histological abnormalities of liver tissues were found to be improved and restored close to the normal group after phthalimide analog treatment (30 mg/kg body weight) for two successive weeks (5 times/week) with potent effect more than thalidomide itself. Therefore, phthalimide analog possessed a potent effect to recover the histopathological changes produced in LI group by the action of CCl_4_.

Malondialdehyde (MDA) is the main end product of lipid peroxidation which is resulted from the induction of ROS by the action of CCl_4_ administration (Plaa, 2000, Ismail et al., 2016). Compared to normal control group, mice in LI model group exhibited marked increase in MDA levels in their liver tissue homogenates. This result agrees with the previously reported studies (Yang et al., 2015, Lu et al., 2017, Lee et al., 2019). Alternatively, the elevated hepatic MDA levels were significantly decreased after the treatment with either phthalimide analog or thalidomide. Phthalimide analog is effectively suppressed the level of MDA in liver tissue homogenates more than thalidomide effect. The elevation in the levels of MDA in liver tissues proposed the oxidative stress augmentation that connected with tissue damage (Dreissgacker et al., 2010). Consequently, the detected antioxidant effect of phthalimide analog against CCl_4_-induced lipid peroxidation in liver tissues may be related to the capability of the analog to suppress the oxidative state that communicated with decrease the lipid peroxidation and consequently diminish MDA levels.

Oxidative stress is a key mediating factor in the progress of hepatic diseases, therefore, reducing the reactions involved in oxidation is a talented therapeutic approach for treatment of liver injuries (Bak et al., 2016). The anti-oxidative enzymes SOD, CAT, and GPx play vital functions in the conversion of active oxygen species into non-dangerous substances. In the present study, CCl_4_-induced LI in the model group showed a decreased antioxidant activity of SOD, CAT, and GPx enzymes in relative to that were found in normal control group. This result is well-matched with the previous reported study that displaying the anti-oxidative enzymes are easily inactivated by ROS during CCl_4_ exposure (Poon et al., 2003). In contrast, mice with injured liver tissues and treated with phthalimide analog, (LI + phthalimide analog) showed a significant improvement in the reduced levels of antioxidant enzymes with remarkable effect more than thalidomide drug. This finding illustrated that phthalimide analog possesses a significant antioxidant activity to guard liver tissues against oxidative damage induced by CCl_4_ through deactivating the oxidative activity of free radicals by rising the antioxidant enzymes activity.

Inflammation is one of the crucial pathological pathways complicated in the induction and development of liver injury (Li et al., 2015, Xu et al., 2019). Thalidomide drug exhibited anti-inflammatory activities in the treatment of various diseases (Kam and Targan, 2000, Hassard et al., 2001). Therefore, the anti-inflammatory activities of new phthalimide analog in comparison to thalidomide were measured through studying its anti-inflammatory effect on the levels of nitric oxide, TNF-α and NF-kB-p65 in liver tissues.

Nitric oxide (NO) is an extremely reactive molecule that formed via inducible nitric oxide synthase (iNOS) and the overproduction levels of NO are complicated in inflammatory responses (Korhonen et al., 2005, Bach et al., 2017, Ning et al., 2018). The present study showed marked elevation in the NO level in liver tissue homogenates of the LI model group, related to normal group. This result is well-matched with the reported data that shows the elevated NO levels in liver tissues of mice injected with CCl_4_ (Khanjarsim et al., 2017, Wu et al., 2017). On the other hand, phthalimide analog diminished the high NO level in comparison to the LI group, with inhibitory effect higher than thalidomide group. This finding is supported by our previous results which showed a potent reduction effect of thalidomide analogs towards NO level in mice bearing solid tumor and human cancer cell lines (Guirgis et al., 2010, El-Aarag et al., 2014). The maximal suppression activity of phthalimide analog over than thalidomide towards the production of NO could be associated to its potent anti-inflammatory activity.

TNF-α is characterized by an important function as a pro-inflammatory cytokine during oxidative stress and inflammation associated to liver injury (Edwards et al., 1993, Weber et al., 2003, Kiso et al., 2012, Germoush et al., 2018, Utaipan et al., 2018). The present study confirms that the administration of CCl_4_ effectively induced inflammation through enhancement TNF-α production in liver tissues of LI model group. This result is compatible with that mentioned CCl_4_-induced liver injury includes the formation of TNF-α and production of pro-inflammatory mediator NO that mediate hepatic inflammation (Decker, 1990). Moreover, TNF-α and the excess production of NO triggered the hepatotoxicity reactions leading to inflammatory hepatic injury (Morio et al., 2001, Gäbele et al., 2009). The LI + phthalimide analog group, illustrated a significant suppression of TNF-α, compared to LI model group, therefore, the potent ani-inflammatory activity of phthalimide analog more than thalidomide could be attributed to its strong inhibition effect towards TNF-α and NO levels in liver tissues.

NF-kB-p65 possess a vital role in regulating an enormous number of genes involved in inflammatory response (Papaconstantinou et al., 2014). Also, the increasing in NF-kB-p65 expression is joined with the increasing in TNF-α expression in damaged tissue (Melgar et al., 2003). It was stated that NF-kB was triggered by TNF-α through I kappa kinase (IKK) canonical phosphorylation (Ding et al., 2004). Also, NO formation was induced by iNOS mediated by TNF-α signaling. Moreover, NF-kB-p65 augmented the transcription of the gene coding for iNOS and TNF-α proteins (Baeuerle and Henkel, 1994). Consequently, the ability of phthalimide analog to provide an effective aid to heal liver injury could explained by its activity to block various inflammatory pathways (NO, TNF-α, and NF-kB-p65) as well as inhibiting oxidative stress status.

The effect of phthalimide analog on the expression levels of PCNA and VEGF in mice was investigated. The results revealed that both PCNA and VEGF levels were elevated in the liver tissues of mice injected with CCl_4_ compared to normal mice. These outcomes are consistent with the previous published studies (Huang et al., 2018, Rahmani et al., 2019, Mercado-Gómez et al., 2020). On the other hand, the increased levels of PCNA and VEGF were normalized after the treatment with the phthalimide analog. This could be due to the inhibitory activity of the phthalimide analog on the of PCNA and VEGF expression levels. It has been reported that phthalimide analogs exhibited an inhibitory activity towards VEGF Receptor 2 enzyme (Othman et al., 2020). Also, the elevated serum level of VEGF in the mice with liver fibrosis was declined by the treatment with carvedilol (Wu et al., 2019).

Hepatocyte apoptosis plays significant roles in the occurrence and progress of liver diseases. Since most drugs are metabolized by the liver; thus, the liver is susceptible to drug-induced liver injury that are accompanied by hepatocyte apoptosis (Cao et al., 2016). In the present study, the results showed that CCl_4_ administration in mice induced apoptosis of liver cells by significant elevation of caspase-3 protein and inhibition of Bcl-2 protein expression. This finding is parallel to the results that previously reported the increasing in apoptosis level in the intestinal epithelium led to gut inflammation (Andaloussi et al., 2013). On the other hand, these conditions were reversed after treatment either with phthalimide analog or thalidomide drug, whereas the highest effect was related to the phthalimide analog as detected by immunolabeling. These outcomes reveal the strong effect of the phthalimide analog in comparison to thalidomide to alleviate CCl_4_-induced liver injury by inhibition of apoptosis.

## 5 Conclusion

The current study displayed the therapeutic effects of new phthalimide analog in comparison to thalidomide drug in the treatment of liver injury induced by CCl_4_ in mice. Phthalimide analog increased the activity of SOD, GPx, and CAT while it reduced MDA and NO levels. In addition, the analog diminished the protein expression levels of VEGF, PCNA, TNF-α, and NF-kB-p65 in liver tissues. Moreover, it revealed anti-apoptotic activity by inducing Bcl-2 and suppressing caspase-3 level. Overall, phthalimide analog ameliorates liver injury induced in mice by CCl_4_ through suppressing of oxidative stress, inflammation, and apoptosis.

## 6 Data availability

The data used to support the findings of this study are available from the corresponding author upon request.

## Funding

This research did not receive any specific grant from funding agencies in the public, commercial, or not-for-profit sectors.

## Declaration of Competing Interest

The authors declare that they have no known competing financial interests or personal relationships that could have appeared to influence the work reported in this paper.
